# Reduction of blood C-reactive protein concentration complements the resolution of sputum bacillary load in patients on anti-tuberculosis therapy

**DOI:** 10.3389/fimmu.2022.1005692

**Published:** 2022-09-16

**Authors:** Khalide Azam, Celso Khosa, Sofia Viegas, Isabel Massango, Nilesh Bhatt, Ilesh Jani, Norbert Heinrich, Michael Hoelscher, Stephen H. Gillespie, Andrea Rachow, Wilber Sabiiti

**Affiliations:** ^1^ Direcção de Laboratórios de Saúde Pública, Instituto Nacional de Saúde (INS), Vila de Marracuene, Moçambique; ^2^ Center for International Health – CIHLMU, Munich, Germany; ^3^ Southern Africa TB and Health System Support Project, East, Central and Southern Africa Health Community (ECSA-HC), Arusha, Tanzania; ^4^ Centro de Investigação e Treino em Saúde da Polana Caniço, Instituto Nacional de Saúde (INS), Cidade de Maputo, Moçambique; ^5^ Division of Infectious Diseases and Tropical Medicine, University Hospital, Ludwig-Maximilians Universität München (LMU) Munich, Munich, Germany; ^6^ Partner Site Munich, German Centre for Infection Research (DZIF), Munich, Germany; ^7^ Division of Infection and Global Health, School of Medicine, University of St Andrews, St Andrews, United Kingdom

**Keywords:** tuberculosis, bacillary load, tuberculosis molecular bacterial load assay, C-reactive protein, treatment response

## Abstract

**Background:**

Tuberculosis (TB) is a difficult-to-treat disease requiring the combination of four antibiotics for a minimum of 6 months. Rapid and quantitative biomarkers to monitor treatment response are urgently needed for individual patient management and clinical trials. C-reactive protein (CRP) is often used clinically as a rapid marker of inflammation caused by infection. We assessed the relationship of TB bacillary load and CRP as biomarkers of treatment response.

**Methods:**

Xpert MTB/RIF-confirmed pulmonary TB cases were enrolled for treatment response assessment in Mozambique. Treatment response was measured using the Tuberculosis Molecular Bacterial Load Assay (TB-MBLA) in comparison with standard-of-care Mycobacterium Growth Indicator Tube (MGIT) culture at baseline and at weeks 1, 2, 4, 8, 12, 17, and 26 of treatment. Blood CRP concentration was measured at baseline, week 8, and week 26. Treatment response was defined as increase in MGIT culture time to positivity (TTP), and reduction in TB-MBLA-measured bacillary load and blood CRP concentration.

**Results:**

Out of the 81 screened presumptive TB cases, 69 were enrolled for 6-month treatment follow-up resulting in 94% treatment completion rate. Four participants did not complete TB treatment and 22 participants had missing CRP or TB-MBLA results and were excluded from TB-MBLA-CRP analysis. The remaining 43 participants—median age, 31 years old [interquartile range (IQR): 18–56]; 70% (30/43) male; and 70% (30/43) infected with HIV—were considered for analysis. Culture TTP and bacillary load were inversely correlated, Spearman’s *r* = −0.67, *p* < 0.0001. Resolution of sputum bacillary load concurred with reduction of blood CRP, *r* = 0.70, *p* < 0.0001. At baseline, bacillary load had a median (IQR) of 6.4 (5.5–7.2), which reduced to 2.4 (0.0–2.9) and 0.0 (0.0–0.0) log_10_ CFU/ml at months 2 and 6 of treatment, respectively. Correspondingly, blood CRP reduced from 1.9 (1.6–2.1) at baseline to 1.3 (0.9–1.7) and 0.4 (0.1–0.8) log_10_ mg/dl at months 2 and 6 of treatment, respectively. CRP reduction trialed bacteriological resolution at a rate of −0.06 log_10_ mg/dl compared to a bacillary load of 0.23 log_10_ CFU/ml per week. Consequently, 14 (33%) and 37 (88%) patients had reduced CRP to normal concentration and bacillary load to zero by the end of treatment, respectively. Pre-treatment CRP concentration and bacillary load, and resolution during treatment were slightly lower in HIV co-infected patients but not significantly different from HIV-uninfected TB patients.

**Conclusion:**

TB-MBLA-measured bacillary load and blood CRP complement each other in response to anti-TB therapy. Slow CRP reduction probably reflects residual TB bacilli in the lung not expectorated in sputum. Combining both measures can improve the accuracy of these biomarkers for monitoring TB treatment response and shorten turnaround time since the results of both assays could be available in 24 h.

## Introduction

Tuberculosis (TB), with an estimated incidence of 10 million people every year, is a major cause of illness and one of the leading causes of death worldwide (1). Before the coronavirus disease 2019 (COVID-19) pandemic, TB was the leading cause of death from a single infectious agent with an estimated 1.2 million deaths among people who are HIV uninfected and an additional 208,000 deaths among people who are HIV infected (1). While efforts have been made to develop effective shorter regimens leading WHO to recommend two new different 4-month regimens to treat TB, the current methods to monitor treatment continue to be a challenge for case management. For instance, the use of clinical examination (classic symptoms of TB such as cough, sputum production, fever, and weight loss) and chest radiography is not accurate and requires backup by effective laboratory tests since different respiratory conditions present with similar clinic signs. Sputum smear microscopy is less sensitive (2), and does not allow species identification or differentiation between viable and nonviable mycobacteria that can lead to determining poor outcome falsely (3). Sputum culture is more sensitive than smear microscopy (2), but is time-consuming, is prone to contamination by rapidly growing microorganisms, takes several weeks to get a result, and requires expensive high-containment laboratories (4). Moreover, culture conversion at month 2 of treatment is not equivalent to treatment success, especially for patients with multidrug- and extensively drug-resistant TB (5, 6). The need of alternative biomarkers, sensitive, specific, safe, and rapid, are urgently needed to monitor TB treatment response.

Recent reports have demonstrated that the Tuberculosis Molecular Bacterial Load Assay (TB-MBLA) VitalBacteria™ has potential to replace culture on monitoring bacterial load during TB treatment (7–9). The 2018 global tuberculosis report acknowledged TB-MBLA as a candidate to replace smear microscopy and culture for monitoring treatment response (10). TB-MBLA is a real-time reverse transcriptase quantitative polymerase chain reaction (RT-qPCR) that uses abundant 16S-rRNA as a target to quantify viable *Mycobacterium tuberculosis*, and measure change in bacillary load as a patient responds to treatment (8, 11). It is unaffected by contamination of other microorganisms, and the result is available in 4 h after the sample reaches the laboratory (11). On the other hand, C-reactive protein (CRP), a protein that rapidly and dramatically increases in the blood in response to inflammation, cell damage, or tissue injury (12), has strong potential to facilitate systematic screening for active TB including in people living with HIV due to its higher sensitivity than clinical examination and radiography (13).

In this prospective study, we investigated the relationship between CRP and TB-MBLA-measured sputum bacillary load as biomarkers of treatment response in patients with active TB and their potential for enhancing treatment monitoring. We show a strong correlation between the two biomarkers in responding to anti-TB treatment that could be exploited as a rapid and accurate composite biomarker for monitoring TB treatment in both low- and high-resource clinical settings.

## Materials and methods

### Patient enrolment and clinical samples

From 17 June 2014 to 28 May 2015, adult (>/=18 years old) presumptive TB cases, able and willing to give informed consent to study participation, including HIV testing, were screened for a diagnosis and treatment response monitoring study at Mavalane Health Centre, Maputo city, Mozambique. Patients who had been treated in the past 6 months or abandoned TB treatment at any time point in the past, those suffering from a condition likely to lead to uncooperative behavior like psychiatric illness or alcoholism, and patients with Karnofsky index below 50% were excluded.

Patients were trained on how to produce quality sputum and asked to bring early morning sputum expectorated after waking up. On arrival at the clinic, spot sputum sample was expectorated before intake of the day’s study drug. Of the two sputa (early morning and spot), the laboratory team selected the high-quality sputum to use for culture and TBMBLA. All participants provided at least 4 ml of sputum (3 ml for culture and 1 ml for TB-MBLA) in each study visit before treatment initiation (baseline) and during treatment (weeks 1, 2, 4, 8, 12, 17, and 26) for testing at the National TB Reference Laboratory. On the same visits, approximately 15 ml of blood was collected from the participants for hematology, HIV, CD4 count, and biochemistry at *Centro de Investigação e Treino em Saúde da Poland Caniço*.

### Mycobacterium growth indicator tube culture

High-quality sputum samples (viscous or mucoid) were homogenized through vortex in 50-ml Falcon tubes containing three to four glass beads. Three milliliters of the homogenized sputum was aliquoted and decontaminated with NALC-NaOH (4% NaOH; 1% NALC; 1.45% sodium citrate) followed by resuspension in 2 ml of phosphate buffer (pH 6.8). To detect growth and quantify bacterial load using culture time to positivity (TTP), 500 µl of the suspension was inoculated into a 7-ml BBL MGIT tube and incubated at 37°C in the BD BACTEC MGIT 960 system (Becton, Dickinson and Company, USA) until any growth in the tube was detected automatically by flagging positive and providing a TTP in days and hours of incubation or flagging negative if no growth was detected after 42 days of incubation.

### TB molecular bacterial load assay

A fraction of the homogenized sputum sample was used for TB-MBLA. One milliliter of each sputum was preserved in 4 ml of the mixture containing 50% guanidine thiocyanate (GTC, Promega, UK), 0.1 M Tris–HCl pH 7.5, and 1% β-mercaptoethanol v/v (Sigma Aldrich, UK) and then stored at −80°C until TB-MBLA was performed. RNA extraction and reverse transcriptase quantitative PCR (RT-qPCR) were performed as previously described (8, 14, 15). The standard curves for translating quantitative cycles (Cq) into bacterial load were performed according to the manufacturer’s guideline (VitalBacteria ™). An RNA extraction control, a positive control (high- and low-concentration *Mycobacterium bovis* Bacillus Calmette Guerin—BCGNCTC5692), a DNA removal control, and a negative (no template) control were included in each run of the assay.

### C-reactive protein measurement

Concentrations of blood CRP of the participants were immunologically measured before treatment (baseline) and at weeks 8 and 26 of TB treatment using The Cobas c111 instrument (Roche Diagnostics Ltd., Switzerland), a continuous random-access analyzer intended for the *in vitro* determination of clinical chemistry and electrolyte parameters. All samples, reagents, calibrators, and controls were used in the instrument according to the manufacturer’s instructions. Measurements of CRP concentration were presented in mg/dl and normal CRP concentration was considered at <1 mg/dl.

### Ethical approval

The study protocol was approved by *Comité Nacional de Bioética para Saúde* (Ref. 274/CNBS/13), Maputo, Mozambique, the Ethics Commission of *Ludwig-Maximilians Universität*, Munich, Germany, and the Teaching and Research Ethics Committee of University of St Andrews University, Scotland, United Kingdom. Only consenting participants were screened and enrolled into the study.

### Statistical analysis

Statistical analysis was performed using GraphPad Prism v.9 (GraphPad Software, California, USA). Treatment response was defined as the fall in bacterial load measured by TB-MBLA, fall in blood CrP level, and/or increase in time MGIT culture positivity. TB-MBLA data were log transformed from large absolute numbers to simplify scaling of graphs. Given that most of the data were not normally distributed, non-parametric methods such medians and interquartile (ranges) were used to describe them. The correlation between TB-MBLA, CRP measurements, and MGIT, and their differences were analyzed using Spearman’s correlation coefficient. Rate of bacillary load or CrP resolution was calculated in Excel using the SLOPE function. Mann–Whitney *U* test was used to calculate the difference in bacillary load or CrP concentration from different underlying conditions such as HIV, age, body mass index, and weight. Multivariable regression was performed to explore the associations between bacillary load as the dependent variable and other clinical parameters. Probability values of <0.05 were considered statistically significant.

## Results

### Study participants

**Figure 1:**
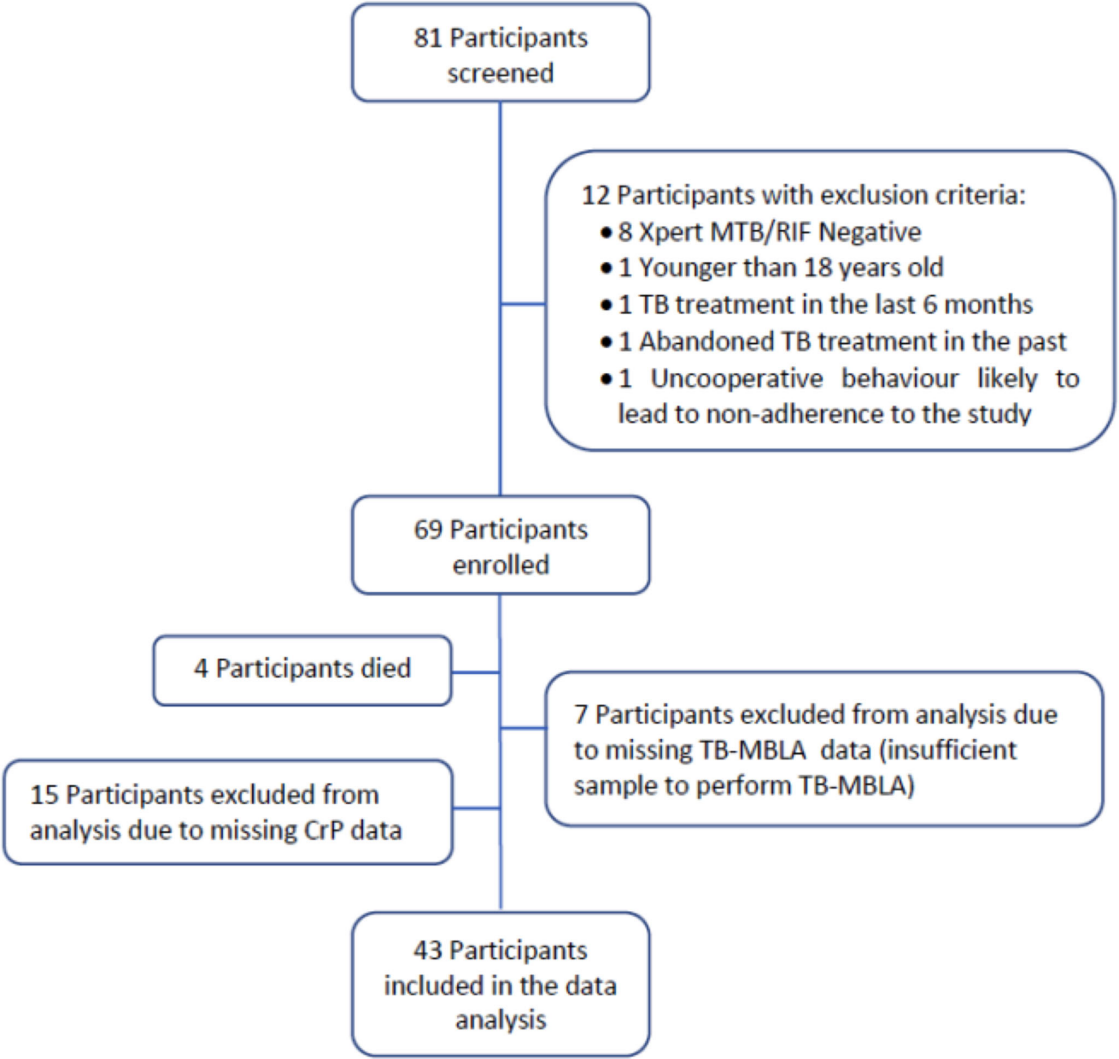
Participant flow diagram.

A total of 81 adult presumptive TB cases were screened and 69 were confirmed as having active TB by Xpert MTB/RIF assay, of which 65 completed treatment and follow-up. Twenty-two participants with missing TB-MBLA data at baseline and/or CRP data at any time point were excluded, leaving 43 who were included in this TB-MBLA-CRP analysis (**Figure 1**).

Among the 43 participants, 70% (30/43) were male and 70% (30/43) were HIV infected. The median age was 31 years old (IQR 18–56) and only 5% (2/43) of the participants reported previous TB history. All participants were treated with the anti-TB and TB/HIV therapy according to the National TB Program guidelines. At baseline, 63% (27/43) of the participants had severe elevation (more than 50.0 mg/dl) of CRP concentration varying from 57.2 to 322.3 mg/dl (**Table 1**).

**Table 1:** Baseline demographic and clinical characteristics of the study participants included in the analysis.

Characteristics at baseline	Participants
**Age in years** Median (IQR)	31 (18/56)
**Sex** Male: % (*n*/*N*)	70 (30/43)
**Race** African: % (*n*/*N*)	100 (43/43)
**Previous TB history: % **(*n*/*N*)	5 (2/43)
**TB drug susceptibility test** Susceptible: % (*n*/*N*) Rifampicin resistant: % (*n*/*N*)	91 (39/43) 9 (4/43)
**CRP concentration** Normal (0 to 1.0 mg/dl), % (*n*/*N*) Marked elevation (10.1 to 49.9 mg/dl), % (*n*/*N*) Severe elevation (≥50.0 mg/dl), % (*n*/*N*)	2 (1/43) 35 (15/43) 63 (27/43)
**HIV status** Infected: % (*n*/*N*)	70 (30/43)
**CD4 count** Male (<200 cells/mm^3^), % (*n*/*N*) Female (<200 cells/mm^3^), % (*n*/*N*)	50 (10/20) 40 (4/10)
**Haemoglobin median** Male, g/dl (IQR) Female, g/dl (IQR)	11.6 (8.3–14.6) 10.5 (8.1–14.1)
**Body mass index (BMI) median** Male, median (IQR) Female, median (IQR)	18.3 (15.3–25.0) 20.0 (14.8–23.5)

### TB-MBLA-measured bacillary load is inversely correlated with MGIT culture time to positivity

**Figure 2:**
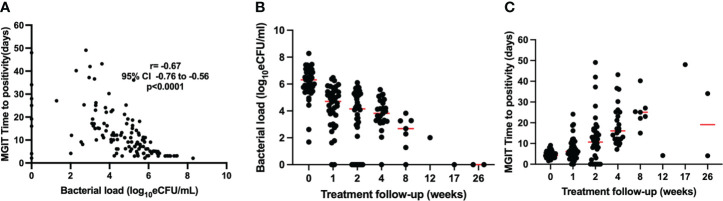
Correlation of bacterial load measured by TB-MBLA and MGIT TTP over treatment follow-up **(A)**. TB-MBLA had a lower variation from the median **(B)** than MGIT TTP for which variation from median increased with treatment and many data points were censured due to contamination **(C)**.

Valid MGIT TTP was determined as time to culture positivity in the absence of contamination (any growth other than MTB). Thus, 146 MGIT cultures from baseline visit, and weeks 1, 2, 4, 8, 12, 17, and 26 had a valid TTP, 11.0 (5.0–18.0) days. MGIT TTP was inversely correlated with the bacterial load measured by TB-MBLA, Spearman’s *r* = −0.67, *p* < 0.0001 (**Figure 2A**). The median (IQR) TTP increased from 3.5 (3.0–4.8) days before treatment to 23 (14–32) and 22 (7–48) at months 2 and 6 of treatment, respectively. Correspondingly, the overall bacterial load of the 146 samples was 2.8 (0.0–4.8) log_10_ estimated colony-forming units (eCFU)/ml. At baseline, bacillary load was 6.4 (5.7–7.1) log_10_ eCFU/ml, declining to 2.4 (0.0–3.0) and 0.0 (0.0–0.0) eCFU/ml at months 2 (week 8) and 6 (week 26) of treatment, respectively. Unlike TB-MBLA, missing data due to invalid TTP and variation from the median increased with time on treatment in MGIT culture (**Figures 2B, C**).

The number of patients with MGIT TTP result decreased from 32 to 9 by week 26 of treatment (**Table 2**).

**Table 2:** MGIT TTP result from baseline to week 26 of treatment.

	Baseline (week 0)	Week 1	Week 2	Week 4	Week 8	Week 12	Week 17	Week 26
MGIT TTP: Median (IQR) days	3.5 (3–5)	8 (6–11)	13 (9–17)	15.5 (11–25)	23 (14–32)	19 (8–32)	13 (5–28)	22.0 (7–48)
No. of samples	32	29	33	28	15	6	7	9

Later stages of treatment were characterized by fewer patients with valid TTP.

### Reduction of blood CRP mirrors resolution of sputum TB bacillary load

We assessed the correlation between the concentration of CRP and the bacterial load measured by TB-MBLA. **Table 3** shows the fall in bacterial load measured at weeks 0, 1, 2, 8, 12, 17, and 26 and CrP measured at weeks 0, 8, and 26 of treatment. All 58 patients had a TB-MBLA result at baseline and at weeks 1, 2, 4, and 26, and 57 patients at weeks 8, 12, and 17 had increasingly fewer data points for MGIT TPP in **Table 2** above.

**Table 3:** The fall in TB bacillary load and CrP over treatment course.

	Baseline (week 0)	Week 1	Week 2	Week 4	Week 8	Week 12	Week 17	Week 26
Bacterial load: median (IQR) log_10_ CFU/ml	6 (6–7)	5 (4–6)	4.5 (4–5)	3 (3–4)	2.4 (0–3)	0.0 (0–3)	0.0 (0–0)	0.0 (0–0)
CrP: median (IQR) mg/dl	76 (42–126)	-	-	–	21 (9–46)	-	-	2.5 (1–6)

TB-MBLA maintained a consistent readout throughout treatment. CrP was only measured at baseline, week 8, and week 26.

**Figure 3:**
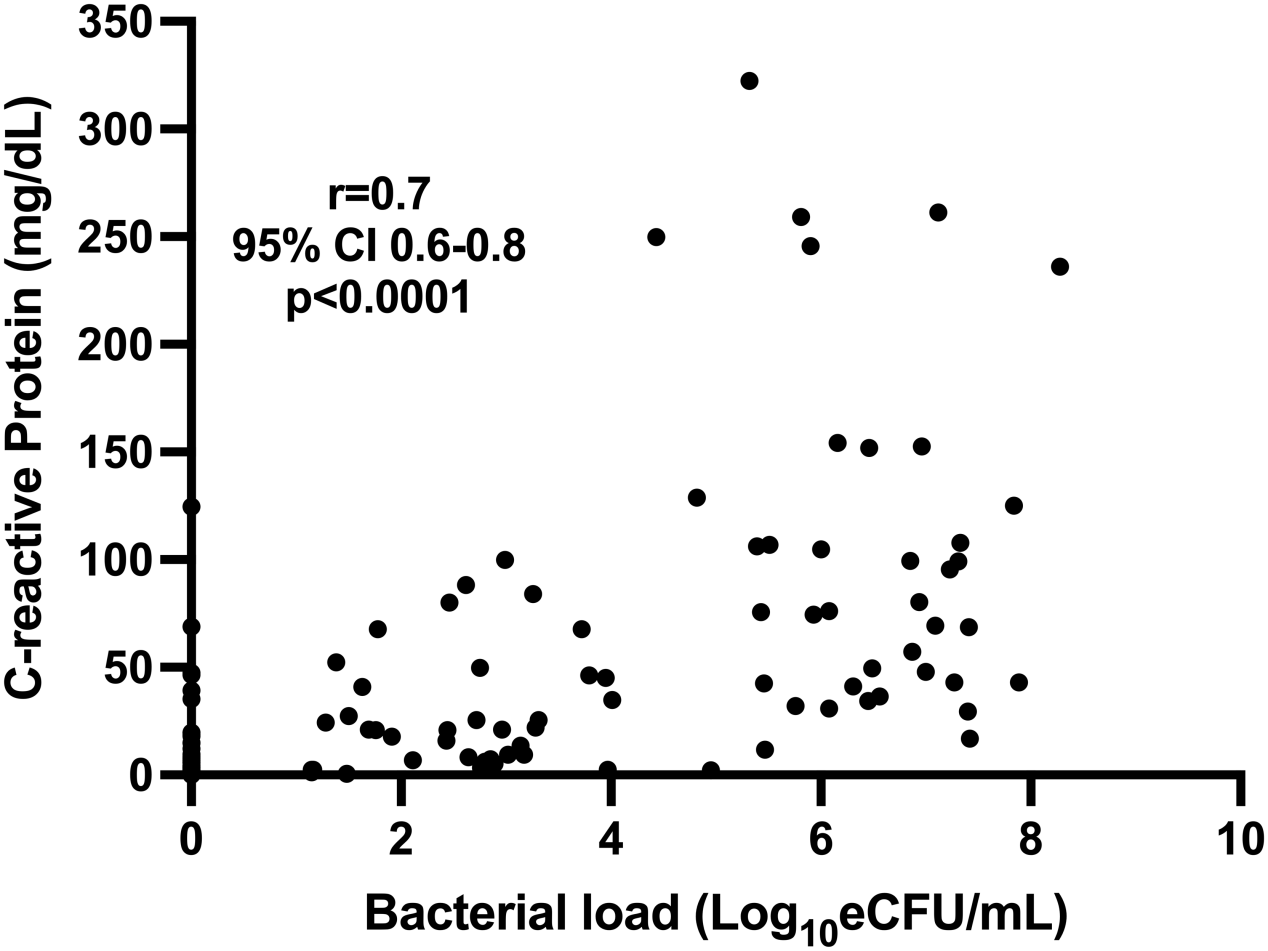
Correlation of blood CRP and sputum TB bacillary load measured by TB-MBLA in 43 patients at baseline, month 2, and month 6.

The concentration of CRP was positively correlated with the bacterial load measured by TB-MBLA, Spearman’s *r* = 0.73 (0.65–0.79), *p* < 0.0001, indicating that a higher CRP (**Figure 3**) was associated with a higher bacterial burden.

**Figure 4:**
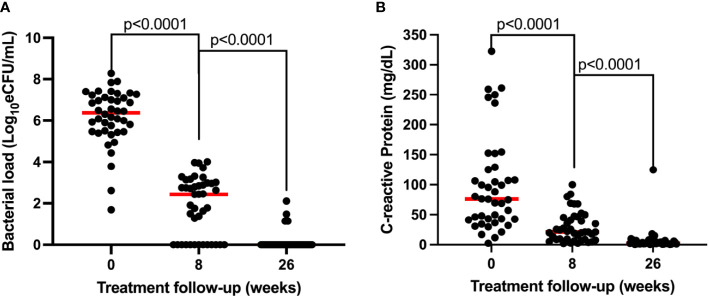
Comparison of the resolution of bacillary load measured by TB-MBLA **(A)** and resolution of blood CRP **(B)** in response to anti-TB therapy observed in 43 patients. Dots represents individual measurements of the variables before (week 0) and at weeks 8 and 26 of the treatment period.

Both sputum bacillary load and CRP declined in response to anti-TB therapy in a manner that mirrored each other. Bacillary load declined from median (IQR) 6.38 (5.47–7.15) at baseline to 2.44 (0.00–2.99) and 0.00 (0.00–0.00) log_10_ eCFU/ml at weeks 8 and 26 of treatment, respectively (**Figure 4A**). Correspondingly, CRP declined from 75.91 (42.17–126.1) at baseline to 21.05 (8.57–45.46) and 2.52 (1.29–6.19) mg/dl at weeks 8 and 26 of treatment, respectively (**Figure 4B**). In both biomarkers, an over 50% reduction within 8 weeks of treatment could be observed. Using Mann–Whitney, medians between pre-treatment and treatment points were compared and found to be significantly different, *p* < 0.0001 for both biomarkers.

### Resolution of CRP trailed bacillary load

**Figure 5:**
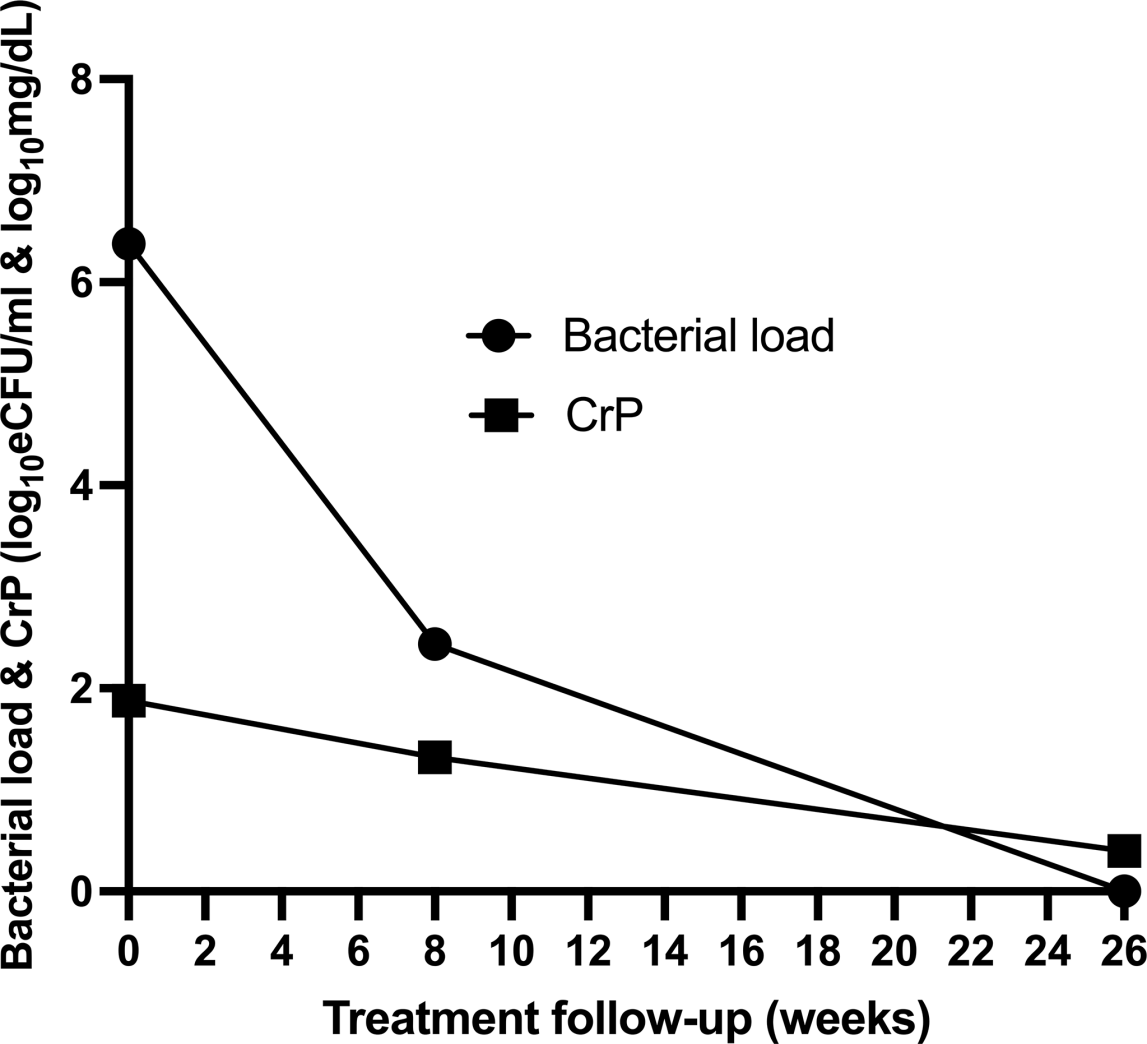
Comparison of the rate of resolution of bacillary load compared to CRP of 43 patients during anti-tuberculosis therapy.

Using SLOPE where *y* = bacillary load or CRP and *x* = time on treatment, we assessed the rate at which the two biomarkers resolved. For consistency, CRP values were log transformed. Bacillary load declined at a rate of −0.23log_10_ eCFU/ml compared to CRP −0.06log_10_ mg/dl per week. Consequently, 37 (88%) patients had reduced bacillary load to zero compared to 14 (33%) who resolved CRP to normal concentration (<1 mg/dl) by the end of treatment at week 26 (**Figure 5**).

### CRP and bacillary load level resolution are independent of HIV coinfection

**Figure 6:**
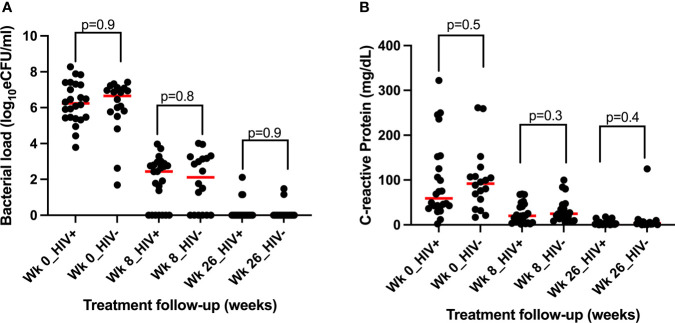
Assessment of the impact of HIV status on the resolution of TB bacillary load measured by the TB-MBLA **(A)** and on the concentration of blood CRP **(B)** resolution among 43 tuberculosis patients before (week 0) and at weeks 8 and 26 of the treatment period.

We assessed whether HIV coinfection had an influence on bacterial load and CRP resolution during treatment. Patients were divided according to HIV status, and their bacterial load and CRP concentration at each sampling point are compared using Mann–Whitney test. At baseline, the median bacterial load, 6.7log_10_ eCFU/ml, of HIV-uninfected patients was slightly higher than HIV-infected patients’ bacterial load, 6.2log_10_ eCFU/ml, but the difference was not significant, *p* = 0.9. By week 8 of treatment, HIV-uninfected patients had a lower bacillary load than HIV-infected patients, indicating a faster rate of clearance. By completion of treatment at week 26, only two cases in each group had a positive bacillary load (**Figure 6A**). Likewise, HIV-uninfected patients had a slightly higher CRP response at both baseline and week 8, which leveled off at week 26 of treatment (**Figure 6B**). In both cases, the differences were not statistically significant.

**Figure 7:**
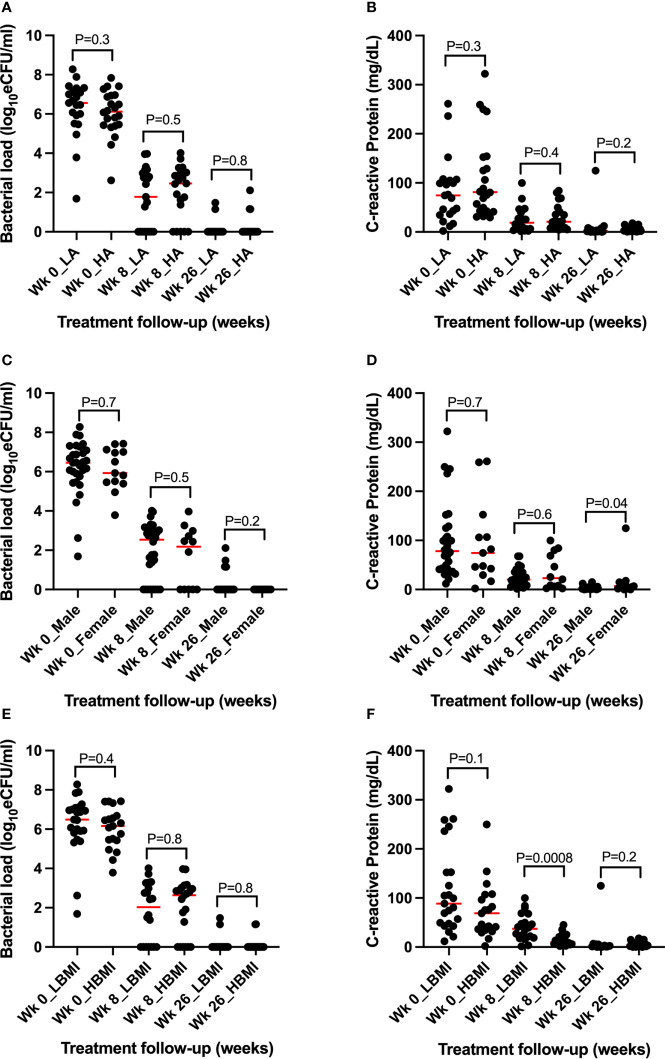
Assessment of the role of demographic parameters in TB bacillary load and CRP concentration and resolution before and during treatment. **(A, B)** Assessment of age revealing no association with bacillary load and CRP concentration and clearance during treatment. **(C, D)** Assessment of gender showing no association with bacterial load but significant difference at week 26 between female and male CRP concentration. **(E, F)** Assessment of BMI showing no association with bacillary load but significant high CRP among the high BMI participants at week 8 of treatment. WK = week, L = low, H = high, BMI = body mass index, Wk 0 = baseline (pre-treatment).

We further explored whether demographic factors such as age, gender, and body mass index (BMI) can explain the difference in TB bacterial load and CRP levels and resolution during treatment. Participant age and BMI and/or male or female were divided into high and low based on median. The difference in the medians of bacterial load and CRP concentration among the high or low groups was tested using Mann–Whitney *U* test. There was no difference in bacterial load resolution in the high/low age groups, and between male and female gender. In contrast, there was a difference in CRP concentration of high BMI, *p* = 0.0008, and being female, *p* = 0.04, at months 2 and 6 of treatment, respectively (**Figures 7A–F**).

We further tested the associations at all time points (baseline, week 8, and week 26) with a multiple regression model using either bacterial load or CRP as a dependent variable. In the first model with bacterial load as a dependent variable, strong association was observed with CRP, *p* = 0.004, and no association was found with age, weight, BMI, and CD4 count (surrogate for HIV status). A similar association with bacterial load was retained when CRP was used as a dependent variable. Interestingly, a weak association, *p* = 0.044, was found between CRP and BMI. Like bacillary load, none of the other patient characteristic parameters were associated with CRP.

## Discussion

This study provides the first comparison between two biomarkers (TB MBLA and CRP) for monitoring TB treatment outcome in patients in a 6-month treatment follow-up. We demonstrated that at treatment initiation, when TB bacillary load is high, it takes a shorter time to result for the standard-of-care MGIT culture. As treatment progress, bacilli are killed and TB-MBLA-measured bacillary load falls and MGIT TTP increases. This inverse correlation has been described in many other studies, indicating that TB-MBLA is comparable with MGIT culture (7, 11, 14). We also showed that TB-MBLA is a powerful method that can be used to monitor TB bacillary load during treatment with the advantage of providing results rapidly (7, 11, 14). It is important to note that MGIT is frequently compromised by contamination, which invalidates TTP data, which compromises the utility of MGIT as a quantitative measure of treatment response. Time to result in MGIT culture is dependent on the sample bacillary load implying that low bacillary load patients will take longer to get results than high bacillary load patients irrespective of whether they reported at the clinic at the same time. TB-MBLA and CRP time to result is independent of the patient bacillary load, which increases the utility profile of the two biomarkers for treatment response monitoring.

Secondly, we demonstrated that CRP has a consistent correlation with bacillary load measured by TB-MBLA. Before treatment, when sputum bacillary load is high, the concentration of blood CRP is also high. This finding concurs with the study that demonstrated pre-treatment higher CRP concentration in patients with pulmonary TB than those with other types of TB disease (16). We show that as patients progress on treatment, both sputum bacillary load and CRP decline in a manner that mirror each other. However, the rate at which CRP was resolved was slower than bacillary load resolution. Consequently, by week 26 of treatment, when most of the sputum bacillary load was undetectable (zero), only 33% had their CRP within the normal interval. The slow resolution of CRP reduction may reflect residual TB bacilli in the lung not expectorated in sputum (17, 18). Alternatively, the presence of ongoing unresolved inflammation in the patient in the absence of live organisms has been shown previously (19). Another hypothesis that may explain these observations is the fact that reduced lung function is high in the first 6 months after diagnosis of tuberculosis, and persists after treatment completion, reaching stability in 13 to 18 months (20). A study by Plit et al. (21) demonstrated that at the end of TB treatment, the elevated concentration of CRP correlates negatively and significantly with decreased lung function (airway obstruction) measured by the Forced Expiratory Volume in one second (FEV1), independent of smoking status. In the same group of patients from our study, we found that 68.9% had lung impairment and suffered from pulmonary obstruction at week 26 (22).

There are few studies of CRP as a measure of TB treatment response. Most studies have focused on investigation of the accuracy of CRP for diagnosis of TB, notably in people living with HIV. In this regard, a recent meta-analysis has shown that in TB patients infected with HIV, CRP has high sensitivity and moderate specificity for active pulmonary TB, suggesting that CRP could be used for screening of active TB (13). Furthermore, a study conducted in Uganda to determine the diagnostic performance of CRP as a triage test for TB among HIV-uninfected patients found that CRP had a high sensitivity and moderate specificity (23), but both lower than 90% and 70%, respectively, as per the WHO recommendation for new diagnostic tests (24). In this study, we did not find significant differences on bacterial load and blood CRP resolution in patients infected and uninfected with HIV. This observation needs further investigation in a large cohort because, ideally, HIV-uninfected people are expected to mount strong inflammation.

BMI below the median of the cohort and being female were associated with high CRP at months 2 and 6 of treatment, respectively, implying a low rate of CRP resolution under these factors. Further research with larger cohorts is required to examine the role of demographic factors, TB/HIV coinfection in resolution of bacterial load and CRP during TB treatment. These studies will further verify the accuracy of CRP as a diagnostic and treatment monitoring tool for TB and the utility of combining it with TB-MBLA. Additionally, larger-scale studies involving more frequent sampling and patients from different geographical and ethnic backgrounds are needed to further test the robustness of this relationship and the possibility to translate it into a composite biomarker for treatment response monitoring in TB. Although CRP is non-specific to TB infection, data from several studies in different populations demonstrating consistency of association with specific biomarkers like TB-MBLA and TB culture may qualify CRP as a stand-alone point-of-care treatment monitoring tool for TB.

We note that our study had several limitations. Firstly, the sample size of 43 participants is not sufficient to make conclusions that can change clinical practice. Fewer sampling points for CRP (baseline, week 8, and week 26) could aid analysis of what was going on during other periods of treatment response and modeling of treatment response dynamics based on CRP. Anti-retroviral treatment (ART) data were missing for HIV-infected patients and this limited analysis of whether ART treatment influences CRP resolution. Nevertheless, we believe that this study has provided initial evidence of the potential utility of CRP in conjunction with TB-MBLA as a rapid (4-h) guide to patient management and may represent a rapid and accurate biomarker of TB treatment response. Crucially, our study has raised important questions for which further research can be premised.

## Data availability statement

The raw data supporting the conclusions of this article will be made available by the authors, without undue reservation.

## Ethics statement

The study protocol was approved by Comité Nacional de Bioética para Saúde (Ref. 274/CNBS/13), Maputo, Mozambique, the Ethics Commission of Ludwig-Maximilians Universität, Munich, Germany and the Teaching and Research Ethics Committee of University of St Andrews University, Scotland, United Kingdom. The patients/participants provided their written informed consent to participate in this study. We confirm that the study was conducted in accordance with the ethics guidelines and regulations.

## Author contributions

AR, KA, NB, and WS conceived the study design. AR and WS trained the clinical and laboratory study team. IM, CK, and KA participated in the data collection for the study. AR, IJ, MH, NB, SG, SV, and WS provided general supervision during the study and reviewed the manuscript. AR, KA, and WS analyzed the data. KA drafted the manuscript. NH, MH, and SG secured the funding from the agencies. All authors contributed to the article and approved the submitted version.

## Funding

This study was conducted under the PanACEA Biomarkers Expansion (PanBIOME) programme and the establishment of the Maputo Tuberculosis Trial Unit (MaTuTU Project), which was funded in parts through the European and Developing Countries Clinical Trials Partnership (EDCTP), PZA study, and the Federal Ministry of Education and Research (BMBF), Germany.

## Acknowledgments

The authors express their gratitude to the study team from the Mozambique National TB Reference Laboratory and TB clinic during the establishment of the Maputo Tuberculosis Trial Unit (MaTuTU) in Mavalane Health Center–Instituto Nacional de Saúde (INS). Special thanks go out to all study participants.

## Conflict of interest

WS and SG provide *pro bono* services to LifeArc, a commercial charity with a contract to commercialize TB-MBLA. This work was performed before University of St Andrews engagement with LifeArc, and the company has had no role in the writing of or the decision to publish this manuscript.

The remaining authors declare that the research was conducted in the absence of any commercial or financial relationships that could be construed as a potential conflict of interest.

## Publisher’s note

All claims expressed in this article are solely those of the authors and do not necessarily represent those of their affiliated organizations, or those of the publisher, the editors and the reviewers. Any product that may be evaluated in this article, or claim that may be made by its manufacturer, is not guaranteed or endorsed by the publisher.
